# Inauguration of the Cameroonian Society of Human Genetics

**DOI:** 10.4314/pamj.v3i1.52447

**Published:** 2009-10-20

**Authors:** Ambroise Wonkam, Marcel Azabji Kenfack, Jude Bigoga, Blaise Nkegoum, Wali Muna

**Affiliations:** 1 Faculty of Medicine and Biomedical Sciences-University of Yaoundé I, Cameroon,; 2 Division of Human Genetics, Institute for Infectious diseases and Molecular Medicine, Faculty of Health Sciences, University of Cape Town, South Africa,; 3 Biotechnology center, Faculty of Sciences, University of Yaoundé I- Cameroon.

**Keywords:** Human Genetics, Africa

## Abstract

The conjunction of “hard genetics” research centers, with well established biomedical and bioethics research groups, and the exceptional possibility to hold the 6th annual meeting of the African Society of Human Genetics (AfSHG, 13th–15th March 2009) was an excellent opportunity to get together in synergy the entire Cameroonian “DNA/RNA scientists” . This laid to the foundation of the Cameroonian Society of Human Genetics (CSHG) that was privilege to hold its inaugural meeting in conjunction to the 6th annual meeting of the AfSHG. The theme was "Human Origin, Genetic Diversity and Health”. The AfSHG and CSHG invited leading African and international scientists in genomics and population genetics to review recent data and provide an understanding of the state-of-knowledge of Human Origin and Genetic Diversity. Overall one opening ceremony eight session, five keynote and guest speakers, 18 invited oral communications, 13 free oral communications, 43 posters and two social events could summarize the meeting. This year’s conference was graced by the presence of one Nobel Prize winner Dr Richard Roberts (Physiology and Medicine 1993). The meeting registered up to ten contributions of Cameroonian scientists from the Diaspora (currently in USA, Belgium, Gambia, Sudan and Zimbabwe). Such Diaspora participation is an opportunity to generate collaborations with home country scientists and ultimately turn the “brain drain” to “brain circulation” that could reduce the impact of the migration of health professional from Africa. Interestingly, the personal implication of the Cameroonian Ministry of Public Heath who opened the meeting in the presence of the Secretary General of the Ministry of Higher Education and a representative of the Ministry of Scientific Research and Innovation was a wonderful opportunity for advocacy of genetic issues at the decision-makers level. Beyond our expectation, a major promise of the Cameroonian government was the creation of the National Human Genome Institute. If this goal comes true, this will be a critical step to bring more genetics for the purpose of Public Health to the Cameroonian people. The sub-Saharan African Region needs significant capacity building in the broad area of basic research in general and Genetics (especially Human Genetics) in particular. In that respect, the existence and current activities of the AfSHG and its impact at the National levels in Africa, is a major development for the continent and an initiative that needs further encouragement from the international community.

## Commentary

After extensive consultation in 2002 and 2003 with researchers in the field of genetics in Africa, United States and Europe, the **African Society of Human Genetics** (AfSHG) was established [1]. A major goal of the society is to organize regular meetings in various parts of Africa to provide a forum for scientists interested in Genetics to meet, interact, network, and collaborate at the national level (2003: Accra, Ghana; 2005: Muldersdrift, South Africa; 2006: Addis Ababa, Ethiopia; 2007: Cairo, Egypt; 2009, Yaoundé, Cameroon). The 5 ^th^ annual meeting was held in Cairo in 2007 in conjunction with the First Annual Meeting of the Division of Human Genetics and Genome Research and the National Society of Human Genetics of Egypt. The 6 ^th^ annual meeting held in Yaoundé emphazised the impact of the AfSHG at the national levels in Africa. Cameroon, with 16 million inhabitants, is frequently referred to as “Africa in miniature” because of its central location on the continent and its many geographical and cultural attributes and human population diversity (there are more than 200 distinct dialects in the country). Sub-Saharan Africa has the highest indices of the global burden of disease and Cameroon is no exception. Even disease patterns have become diverse since populations in this region are living under the double burden of transmissible and chronic diseases. How can recent and significant knowledge in genetics and especially human genetics contribute to alleviate suffering and premature death in this region especially in the current context of increasing poverty? How can we exploit our diversity to further bridge the gap of our health disparities?

Genetics is an evolving field in Cameroon with at least five centers involved in medical genetic research (Service of Genetic Medicine, Gyneco-Obstetric and Pediatric Hospital, Yaoundé), and mostly in molecular biology research on HIV and malaria (the Biotechnology Center (University of Yaoundé I), the Chantal Biya International Reference Research Centre (CIRCB, Yaoundé), the Walter Reed Johns Hopkins Cameroon Program (Yaoundé), and the National Research Institute on Medicinal Plants (IMPM, Yaoundé). The conjunction of these “hard genetics” research centers, with well-established biomedical and bioethics research groups, and the possibility to hold the 6 ^th^ annual meeting of the AfSHG (13 ^th^ –15 ^th^ March 2009 ) was an opportunity to get together in synergy the entire Cameroonian “DNA/RNA scientists” (HIV, Malaria, TB and Human geneticists). The 6 ^th^ annual meeting of the AfSHG then induced the foundation of the Cameroonian Society of Human Genetics. This latter society was thus privileged to host with its inaugural meeting, the 6 ^th^ annual meeting of the AfSHG, an opportunity for Cameroon specifically and Central Africa generally. Hosting this event represents a potent statement of the founder’s desire to engage with genetics and genomics and to use existing continental resources to move forward and to develop capacity to face health-related challenges ahead.

A programmatic highlight of the AfSHG Cairo meeting was the announcement of the African Genome Project (AGP) [2]. Since the Cairo meeting, there has been considerable discussion by African and international scientists and potential funding agencies regarding how to fully engage Africa in genomic science. These discussions led to the development of an International Frontiers meeting in Cameroon following the AfSHG 6 ^th^ annual meeting. The theme of 2009 Yaoundé conference was “Human Origin, Genetic Diversity and Health.” The AfSHG invited leading African and international scientists in genomics and population genetics to review recent data and provide an understanding of the state-of-knowledge of Human Origin and Genetic Diversity. About 120 participants from 12 African countries, Europe, America, and Australia attended the meeting.

It was a great honor that this year’s conference was graced by the presence of one Nobel Prize winner Dr Richard Roberts (Physiology and Medicine 1993). This distinguished scientist delivered the keynote address during the opening ceremony. Overall, we were privileged to have many other eminent scientists participating in our program this year, including Drs. David Bentley (Illumina Inc) and Sir Walter Bodmer (University of Oxford), both longtime supporters of the Society, who delivered keynote addresses. Other invited lectures were presented by Drs. Eric Green (NHGRI, NIH) and Michael Hayden (University of British Columbia). The meeting also had increased participation of African and international students. In addition, the meeting registered up to 10 contributions of Cameroonian scientists from the Diaspora (currently in USA, Belgium, Gambia, Sudan and Zimbabwe). Such Diaspora participation is an opportunity to generate collaborations with home country scientists and ultimately turn the “brain drain” to “brain circulation” concept [3]. The contribution of the AfSHG to such a “brain circulation” could reduce the impact of the migration of health professional from Africa.

The meeting consisted of an opening ceremony, 8 sessions, 5 keynote and guest speakers, 18 invited oral communications, 13 free oral communications, 43 posters, and 2 social events. The medical genetic session was opened by an invited lecture of Prof Michael Hayden entitled “Pharmacogenomics of severe adverse drug reactions - Implications for the African Continent.” The lecture emphazised that adverse drug reactions (ADRs) to medications cause significant morbidity and mortality. In the United States, ADRs rank as the 5th leading cause of death and cost billions of dollars annually in healthcare costs . Children are at an increased risk of severe ADRs. We hypothesized that genetic polymorphisms in drug metabolism genes underlie a significant portion of concentration-dependent ADRs. Prof. Hayden repported activities of the Canadian Pharmacogenomics Network for Drug Safety, a nationwide ADR surveillance network of full-time clinicians across Canada to identify, report, and collect comprehensive case information for severe ADRs to solve key drug safety concerns. It appears that many of the predisposing genetic markers have frequencies in populations of African descent suggesting that side effects to certain drugs may be more frequent in Africans. In addition, measures to reduce the impact of ADRs on the lives of patients are particularly relevant in the African context where some toxic effects may be more prevalent but often go undetected. There is a need to determine the differences in allele frequencies in African subpopulations for genes involved in specific medication as well as the need for case-control studies for adverse drug reactions in patients in Africa. The ultimate goal of such phamacogenomics studies is to reduce medication costs (use medications only in patients who will benefit) and improved medication safety (i.e., avoid serious adverse drug reactions, improved understanding of population differences in susceptibility to ADRs, prevention of disability in communities with less resources).

Presenting a very interactive clinical approach to dysmorphology, Dr Armand Bottani (Geneva University Hospital, Switzerland) showed that trying to reach a definite diagnosis in patients with peculiar physical traits and/or mental retardation is a challenging task in clinical genetics, but a prerequisite one for an adequate genetic counseling. Much information leading to the delineation of a potentially known condition or syndrome can be gathered from a systematic clinical examination, paying particular attention to some key dysmorphic elements. He illustrated some of them and gave an overview on how to proceed as a clinician when facing such patients. In the context, could molecular cytogenetic replace classical chromosomal analysis? That what the question of Dr Frédérique Bena (Geneva University Hospital, Switzerland). She evidenced from literature review that Array-CGH is a powerful tool to detect small chromosomal unbalances < 5 Mb (zoom 100x on chromosomes). Array-CGH may be used as an adjunct to standard cytogenetics testing in the evaluation of patient with mental retardation and/or congenital anomalies. But, Array-CGH should not be used as a first-tier test in prenatal diagnosis. Finally, Dr Ambroise Wonkam (University of Yaoundé I, Cameroon) shared his experience of practicing Medical Genetics in a Sub-Saharan Africa context. The major preliminary trends where raised: 1-prenatal diagnosis of sickle cell anaemia were the principal activity in foetal medicine clinics; 2-Clinical dysmorphology and neurogenetics practice were difficult in the context of limited available genetic analysis; 3- Reproductive medicine consultations were dominated by abnormalities of external genitalia and relatively few infertility consultations; 4- Oncogenetic consultations seemed surprisingly low. He demonstrated that practice of medical genetics involving counseling, prenatal diagnosis, and in some cases medical abortion, is possible in Cameroon. The preliminary experience emphasized the need of international collaborative effort to overcome the lack of human, technical, and financial resources in Cameroon.

Interestingly, the personal implication of the Cameroonian Ministry of Public Heath who opened the meeting in the presence of the Secretary General of the Ministry of Higher Education and a representative of the Ministry of Scientific Research and Innovation was an opportunity for advocacy of genetic issues at the decision-makers level. Beyond our expectation, a major promise of the Cameroonian government was the creation of the National Human Genome Institute. If this goal comes true, it will be a critical step to bring more genetics for the purpose of public health to the Cameroonian people. This statement resounded outside Cameroon during the 16 ^th^ meeting of the South African Society of Human Genetics (SASHG), held 5–7 ^th^ April in Stellenbosch. Indeed in his opening ceremony, the Director General in the Ministry of Science and Technology, Dr Philip Mjwara, recalled the promise of the Cameroonian government and strongly recommended the SASHG to affiliate to the AfSHG in order to improve synergy concerning genetics development in Africa. These sentiments are probably due to South African scientists who were present at the AfSGH Yaoundé meeting.

The sub-Saharan African Region needs significant capacity building in the area of basic research in general and genetics (especially Human Genetics) in particular [4]. In that respect, the existence and current activities of the AfSHG and its impact at the national levels in Africa, is a major development for the continent and an initiative that needs further encouragement from the international community.
